# Directions of Changes in the Health Values of Dairy Products in the Opinion of Consumers

**DOI:** 10.3390/nu13061945

**Published:** 2021-06-05

**Authors:** Marta Sajdakowska, Jerzy Gębski, Krystyna Gutkowska

**Affiliations:** Department of Food Market and Consumer Research, Institute of Human Nutrition Sciences, Warsaw University of Life Sciences (SGGW-WULS), 159C Nowoursynowska Street, 02-787 Warsaw, Poland; jerzy_gebski@sggw.edu.pl (J.G.); krystyna_gutkowska@sggw.edu.pl (K.G.)

**Keywords:** consumer, health values, dairy products

## Abstract

The aim of our research was to assess whether and to what extent the perceived change in the content of selected ingredients in dairy products is important for Polish consumers in accepting the enhancement of the health benefits of dairy products, including yogurt. The data were collected using a CAPI (Computer Assisted Personal Interview) survey on a sample of 983 consumers. The logistic regression model was used to predict the behavior of consumers associated with their willingness to accept the health aspects of improving dairy products. The results indicated that changes in the level of selected ingredients enhanced the willingness to accept increasing the health value of the product. The socio-demographic characteristics of the participants were not associated with the degree of their willingness to accept the improvement of the perceived health attributes. Practitioners in the dairy industry and policy makers can benefit from these results. When designing food products, it is worth focusing on increasing the nutritional value and enhancing the health value of food that is perceived by consumers as generally possessing positive health benefits, rather than on food that is perceived by them as possessing negative qualities.

## 1. Introduction

Consumers perceive health as the most important value, therefore, while making a food choice, health-related motives increasingly influence their decision [1]. Moreover, in recent years, consumer demand for health-enhancing food products (e.g., dairy products) has risen rapidly [2,3,4].

Additionally, some studies have evaluated the health effects of the consumption of dairy products with prebiotics as having positive results for anti-diabetic and anti-hypertensive properties and improving the blood lipid profile [5]. Furthermore, findings show the impact that fermented foods and beverages (e.g., yogurt) produced or preserved through the growth of microorganisms have on general health, mainly on the gut microbiota balance and brain functionality [2]. Research also shows that fermented dairy products have unique qualities owing to the fact that they are excellent ground substances for the incorporation of ingredients and/or nutrients that provide the end product with properties that are beyond solely nutritional [6]. Moreover, food enrichment can prevent or treat some of the diseases, especially in young children and it has a significant impact on improving the health of the community [7].

Yogurt still plays an important role in the human diet today due to its pleasant taste and health benefits [8]. Yogurt is a good source of several micronutrients and may help to improve diet quality and maintain metabolic well-being as part of a healthy and energy-balanced dietary pattern [9]. Furthermore, yogurt consumption in epidemiological studies is associated with a lower body mass index, lower body weight, smaller waist circumference, and lower body fat [10]. Moreover, economic gains from yogurt consumption should not be neglected. Increasing yogurt consumption in the adult population in the UK can result in substantial cost savings to the UK National Health Service as well as in lowering the incidence of type 2 diabetes (T2D), which is beneficial for a patient [11].

On the one hand, consumers tend to display unhealthy nutritional behaviors, which poses a major challenge for public operators to promote healthier food habits. On the other hand, better-educated, more conscious, and sophisticated consumers can contribute to numerous innovations on the food market. Furthermore, the growing information available to consumers also stimulate the search for foods that are not only healthy but also increasingly more personalized [12].

Producers targeted their aims at lowering sodium, fat, and cholesterol contents in food products [13] and on reducing sugar contents; policy makers have put forward numerous recommendations as well [14]. Moreover, to meet consumer demands for healthier options, manufacturers are making, e.g., low-fat and non-fat versions of their most popular flavors of dairy products including yogurts [8].

When looking for food products on the market, including those with an improved composition, consumers use various sources of information and information on the food label [15,16,17]. In general, consumers who are health-conscious with respect to their lifestyle and diets derive high utility values from the nutritional information of the product [18]. Three factors play an important role in innovation in the food sector: health-promoting aspects of dairy products, the significance of food labeling in consumer purchasing decisions, and consumer awareness of the link between diet and health. Therefore, producers should devise a method to reduce unhealthy ingredients in food products and enhance the healthy ones [19].

However, the interest in a product with a changed composition also depends on the acceptance of the product as a carrier of the added ingredient [20]. In addition, some authors point out that consumers see products that are intrinsically healthy as credible carriers of functional messages [21,22] and they show a more positive attitude when the functional ingredient is inherent in the original product [23].

Nevertheless, some evidence from research on preferences for foods in which changes that relate to perceived health benefits have been made (i.e., increasing and/or decreasing levels of certain food components) are contradictory and other research findings do not fully explain the observed behaviors [5,24,25,26,27].

Therefore, the aim of our research was to assess whether and to what extent the perceived change in the content of selected ingredients in dairy products is important for Polish consumers in accepting the enhancement of health benefits of dairy products, including yogurt.

## 2. Materials and Methods

### 2.1. Data Collection Process and Description of Questionnaire

The sample in our study (*N* = 983) was obtained from the address database of Social Security and was representative of the national population in terms of age, gender, and the region that the consumers inhabited. Each of 16 voivodships in Poland were included in the survey. This paper encompasses some of the findings from a larger multidisciplinary study discussing the innovation in products of animal origin and its drivers and obstacles, including consumer perception and the acceptance of innovative food products [28,29,30]. The interviews were carried out at respondents’ homes on a face-to-face basis by a professional market research agency following the ESOMAR (European Society for Opinion and Marketing Research) code of conduct using the CAPI (Computer Assisted Personal Interview) technique. A detailed selection of the research sample is discussed in another paper referring to the conducted research [28,30]. All respondents were aged 21–70. Only those respondents who met the recruitment criteria, i.e., made their own or cooperative food purchases and declared dairy product consumption, participated in the study. The criterion which excluded the surveyed respondents from taking part in the research was the declaration of not buying or consuming dairy products.

The questionnaire used in the study was structured in a few main blocks and covered aspects such as (Supplementary Questionnaire 1):

(1) Declarations on the acceptance of enhancing the health value of dairy products (a question: Do you approve of enhancing the health benefits of dairy products?): (1) yes and (2) no; declarations regarding the acceptance of enhancing the nutritional value of these products (a question: Do you think it is possible to enhance the nutritional value of dairy products?): (1) yes and (2) no;
(2) Opinions on the perceived health benefits of dairy products (a question: Please indicate which of the given/mentioned properties of dairy products you consider to be the most characteristic of dairy products with high health value. Please indicate no more than 4 answers: they (1) have a positive effect on your silhouette, (2) contain no preservatives, (3) contain the addition of live bacteria cultures, (4) have a short shelf life, (5) are processed little, (6) have low levels of selected ingredients, e.g., salt, sugar, and fat, (7) have a high mineral and vitamin content, (8) are recommended by scientific institutions, (9) the packaging carries information indicating its health benefits, e.g., “Contributes to lowering cholesterol levels”, (10) contain no ingredients that may cause allergies, (11) do not contain food colors, and (12) are produced with respect for the rights of farm animals;
(3) Opinions on the nutritional perception of dairy products (a question: Please indicate which of the given /mentioned characteristics of dairy products you consider to be most characteristic of dairy products with high nutritional value. Please indicate no more than 4 answers: they (1) contain a lot of vitamins and minerals, (2) are rich in essential fatty acids, (3) contain a lot of fat, (4) contain a lot of protein, (5) contain a lot of cholesterol, (6) contain a lot of fiber, (7) contain a lot of omega-3 fatty acids, (8) contain live bacteria cultures, (9) have information on the packaging that informs them about the nutritional benefits, e.g., “Rich in protein”, “With increased calcium content”, and “Sugar-free”, and (10) high in calories;
(4) Opinions on increasing the content of selected ingredients in dairy products (a question: Do you think that the content of the following ingredients in dairy products should be increased? The number 1 means “definitely should not be increased” and 7 means “definitely should be increased”): (1) minerals, (2) fiber, (3) cholesterol-lowering ingredients, (4) omega-3 fatty acid, (5) live bacteria cultures, (6) protein, and (7) coenzyme Q10;
(5) Opinions on yogurts with selected characteristics referring to changes in their composition (a question: Would you consider buying the following types of yogurt? Please use a scale from 1 to 7, where 1 means “definitely not” and 7 means “definitely yes”): (1) with increased levels of certain vitamins and minerals to prevent deficiencies, (2) with added food colors (3) with added omega-3 fatty acids, (4) with information on the packaging such as “Helps lower cholesterol”, (5) with a longer shelf life, (6) with information on the package such as “Sugar-free”, (7) with ingredients that may cause allergies removed, e.g., milk and dairy products, (8) with added selenium, (9) thickened by the addition of various ingredients, (10) with reduced levels of certain ingredients, e.g., salt or sugar to prevent various diseases, e.g., obesity, hypertension, and diabetes, (11) with added coenzyme Q10, (12) with added calcium, (13) with added zinc, (14) with added magnesium, (15) with increased levels of substances with beneficial health effects, (16) with a simple message on the package such as “High protein content”, (17) with a reduced level of fat, (18) in which selected ingredients have been replaced with others, e.g., sugar with sweeteners, and (19) with added fiber.

### 2.2. Statistical Analysis

A preliminary analysis of variables characterizing the interest (purchase) in yogurt having certain characteristics prompted a factor analysis to reduce the number of variables. The factor analysis with the applied varimax factor rotation resulted in obtaining 3 factors characterizing the following yogurt properties: factor 1 (features related to the addition of substances to yogurt that enhances its health properties); factor 2 (features related to the reduction in certain ingredients that improve its health properties); factor 3 (features related to the addition of certain ingredients that improve its sensory properties) (Table 1).

Substantially, the scree plot and the eigen value of at least 1 were used to select the number of factors. The obtained factors were normalized using the range method (subtracted minimum and divided by standard deviation/SD). After normalization, factors were given values ranging from 0 to 1. In order to facilitate the interpretation, normalized factor values were assigned to quartiles.

A regression model was used to assess the impact of individual yogurt features on the acceptance of increasing the health value of dairy products. Due to the dichotomous nature of the dependent variable (regressant), a logistic regression model was used.

The independent variables (regressors) indicated for the model (including 3 newly obtained factors) were selected using the stepwise method. Finally, statistically significant variables (V) remained in the model at the significance level *p* < 0.05 (V1—they are characterized by a low level of selected components, e.g., salt, sugar, fat; V2—they have a high content of minerals and vitamins; V3—they do not contain food colors, V4—possibility of increasing the nutritional value of dairy products; V5—they contain a lot of fat; V6—they contain a lot of protein; V7—importance of increasing mineral content; V8—increasing; V9—decreasing).

In the next stage of analysis, an adjusted logistic regression model was developed to determine the effect of selected socio-demographic variables (gender, age, education, and place of residence) on the value of the model coefficients. However, none of the above socio-demographic variables proved to be statistically significant in an adjusted logistic regression model.

In general, there are many indicators that can be used to assess the quality of the models created. High quality of both models was confirmed by c-statistic, in which the value for the unadjusted model was 0.875 and for adjusted model it was 0.892. Fairly high value of Pseudo R-Square for both models (0.387 for unadjusted model and 0.421 for adjusted model) also indicates good fit of the model to the data. Moreover, good fit of both models was also confirmed by Hosmer and Lemeshow Goodness-of-Fit Test (respectively: unadjusted model *p* = 0.7603 and adjusted model *p* = 0.8881).

The statistical analysis was performed using the SAS 9.4 statistical package (SAS Institute, Cary, NC, USA).

### 2.3. Profile of the Total Sample

A slightly higher number of women than men took part in the study. People under the age of 34 accounted for almost 1/3 of all the respondents, while every 10th respondent was aged 65 or more. Half of the respondents had at least secondary education (the sum of secondary and higher). About 2/5 of the respondents came from cities with a size of 20 to 500 thousand residents. The socio-demographic characteristics of the studied sample are presented in Table 2.

## 3. Results

### Willingness to Accept Enhancing Health Values

Some consumers are of the opinion that products characterized by a low level of selected ingredients, e.g., salt, sugar, and fat prove their high health value. Such an opinion increases by 68.3% the probability of acceptance of increasing the health value of dairy products in relation to the opposite opinion (answer: “no”) (OR: 1.68; 95% CI: 1.09–3.32) (Table 3).

The opinion that products with a high content of minerals and vitamins are characterized by high health values and therefore the probability of acceptance of enhancing the health value of dairy products increases by 89% in relation to the opposite opinion (answer: “no”) (OR: 1.89; 95% CI: 1.12–3.19).

The opinion that products that do not contain food colors are characterized by features indicating high health values and therefore the chance of the acceptance of enhancing health values of dairy products decreases by 28.9% in relation to the opposite opinion (answer: “no”) (OR: 0.71; 95% CI: 0.42–0.97).

The consumers who accept increasing the nutritional value of dairy products are over 7 times more likely to accept enhancing the health value of dairy products in relation to their opponents (OR: 7.69; 95% CI: 4.48–13.21).

The consumers who think that a high fat content indicates a high nutritional value of dairy products are 57.7% less likely to accept an increase in the health value of dairy products compared to those who have the opposite opinion (OR: 0.42; 95% CI: 0.21–0.84).

The surveyed participants of the study who think that a high protein content demonstrates the high nutritional value of dairy products are 60.7% more likely to accept an increase in the health benefits of dairy products than those who have the opposite opinion (OR: 1.61; 95% CI: 1.05–2.73).

For the consumers who are of the opinion that the mineral content of dairy products (calcium, selenium, magnesium, and zinc) should be increased, the chance of accepting enhancing the health benefits of dairy products rises by 46.4% per each point of increase in this opinion (scale 1–7) (OR: 1.46; 95% CI: 1.25–1.71).

When the respondents’ interest in purchasing selected yogurts with an increased or decreased level of selected ingredients was significant, it was assessed to what extent it may affect the level of acceptance in terms of their willingness to accept the increase in the health benefits of dairy products.

If the variable “increasing” belongs to the Q2 quartile, the probability of the willingness to accept enhancing the health value of dairy products rises by 69.6% (OR: 1.70; 95% CI: 0.81–3.57) (however, this level is not statistically significant for the model) in relation to the reference level (Q1), with the remaining model variables kept constant. Belonging to Q3 increases this chance 2.79 times (OR: 2.79; 95% CI: 1.34–5.83) and belonging to Q4 increases this chance 4.59 times (OR: 4.59; 95% CI: 2.13–9.91) compared to Q1.

If the variable “decreasing” belongs to the Q2 quartile, the probability of accepting the increase in the health value of dairy products rises by 89% (OR: 1.89; 95% CI: 0.09–4.00) compared to the reference level (Q1). If this variable belongs to Q3, this chance increases almost 2.5 times (OR: 2.43; 95% CI: 1.18–5.00) and belonging to Q4 makes this chance 2.85 times higher (OR: 2.85; 95% CI: 1.37–5.92), evidently, in relation to Q1 of this variable.

## 4. Discussion

Consumers’ demand for healthy food with a pleasant taste observed in recent years resulted in enrichment and fortification of some dairy products. Consequently, the dairy products, including yogurt, gained a pleasant taste and increased health benefits, which attracted new consumer groups [8]. Moreover, owing to its healthy image, yogurt can be appropriately used as a probiotic carrier. It is suggested that a regular intake of probiotic yogurt helps keep a healthy lifestyle, which is essential to retain health and reduce potentially long-term risk of disease [31]. Furthermore, as indicated by findings, health benefits and ingredient naturalness are highly valued, but specific preferences towards them depend greatly on factors such as education, income, and food purchasing decisions; thus, there is a general preference for naturally occurring nutrients over fortification [32]. The logistic regression model presented in the paper was used to predict the behavior of Polish consumers associated with their willingness to accept the health aspects of improving dairy products and highlighted the opportunities for producers in the dairy products market.

### 4.1. The Importance of Socio-Demographic Variables in Increasing and Decreasing Levels of Selected Ingredients

The results of our research indicated that opinions on changes in the level of selected ingredients in dairy products increase the willingness of the surveyed consumers to accept enhancing the health value of the product. This applies in particular to situations where changes in the level of these ingredients are positively perceived by consumers, i.e., increasing the level of ingredients having a beneficial effect on health and lowering the level of ingredients for which its potential presence in the product could have negative health consequences. Results of the studies among European consumers show that they perceive the nutritional value of foods as important when selecting foods and that it is even more important while qualifying (fiber and vitamins/minerals) rather than for disqualifying nutrients (energy, fat, saturated fat, salt, and sugars) [33].

In general, the findings of our research presented in the article indicated that the positive aspect perceived by consumers, connected with increasing and/or decreasing unfavorable components, plays a much greater role in their willingness to accept enhancing health values of dairy products compared to the influence of socio-demographic variables, e.g., gender, age, education, and place of residence. Moreover, as it was indicated previously, none of the above socio-demographic variables proved to be statistically significant in an adjusted logistic regression model. It should also be emphasized that the literature points to the importance of such variables as: gender, age, or education in the choice of food with health benefits. In general, women display more favorable attitudes towards health [34] and they more often show pro-health behaviors in food choice, choice motives, preferences, and food intake [35]. Moreover, better-educated consumers with higher annual incomes and with young children and women, in particular, tend to buy health-promoting food products [36]. Furthermore, some results also show that eating habits are perceived as more serious among younger individuals with a stronger health motive [34]. In the case of (Turkish) diary consumers, there is a difference between younger, better-educated, more affluent, and regular consumers who have more favorable perceptions about milk, cheese, and yogurt; and elderly, less educated, poorer consumers with lower consumption of dairy products who are less in favor of milk and milk products [37]. Nevertheless, research among some groups of consumers (e.g., Italians) show that gender and age are not significantly associated with the degree of knowledge and consumption frequency of functional foods (including dairy products). However, the same study findings confirm that functional food users have often been better-educated [38]. In another piece of research, important factors were identified as having an impact on the consumption of probiotic products. They include education, income, and occupation. The consumers more willing to consume probiotic dairy products were well-educated and possessed higher income levels [39]. The consumption of dairy products with prebiotics was associated with anti-diabetic and anti-hypertensive properties, improvements in the blood lipid profile, immunity, and intestinal health [5]. Other research findings suggest that dairy product intake during pregnancy could, perhaps, have an effect on neonatal head circumference, placental weight, and gestational weight gain [40]. Furthermore, for example, when a particular nutrient is taken into account (e.g., CLA: Conjugated Linoleic Acid), the consumer target segment for most CLA-enriched milk products can be principally characterized as being health-conscious and middle-aged consumers who already believe in the health properties of conventional milk products [41].

### 4.2. The Importance of Perceiving Selected Food Ingredients in the Willingness to Accept Enhancing Health Values of Dairy Products

Results of our research indicate that the positive aspect related to increasing beneficial (mineral components) and/or decreasing harmful (salt, sugar, and fat) food components perceived by consumers plays a greater role in their willingness to accept the enhancing health values of dairy products than socio-demographic variables characterizing respondents. This may be due to the synergistic effect produced by these particular ingredients (i.e., an increase in positive consumer perception of an ingredient in a positively perceived food product). Regarding some ingredients (salt, fat, and sugar) in the food product and their impact on health, findings of other studies showed that consumers were unaware of how much salt they consumed. However, they were aware of the link between the high salt intake and certain negative health consequences, although they were unsure about the precise dependance [42]. Referring to sugar, research shows that although dairy products are usually regarded as healthy, flavored dairy products may significantly contribute to the daily intake of sugars. Therefore, a reformulation of their composition is recommended [43]. In general, decreasing, e.g., obesity or cardiovascular disease can be achieved by eating healthier foods with reduced contents of fat, salt, and sugar [44]. Our research also indicated that increasing the levels of certain minerals in dairy products may be viewed positively by consumers because the above mentioned minerals are generally understood to have health benefits (e.g., calcium is a valuable bone builder), which is supported by other research [45,46]. Moreover, in general, consumers may attribute great importance to the components that they know well and are confident about their impact on health [47]. Furthermore, other research showed that consumers with high self-control are more likely to hold beliefs and intentions to participate in future healthy behavior. Additionally, some consumers with high self-control are more likely to present healthy intentions and to reduce unhealthy intentions [48].

Our research showed that in the context of the perception of the nutritional value of dairy products and in relation to nutrients such as fat and protein, which according to the surveyed consumers can determine the high level of nutritional value of the dairy product, consumers’ declarations were quite unambiguous. A high level of fat, as a feature describing products with high nutritional value, resulted in a decrease in the willingness to accept enhancing the health benefits of such a product. On the other hand, the high amount of protein in the product determined the positive perception of the product, i.e., it was evaluated as a product of high nutritional value and, thus, the level of willingness to accept the increase in health values of such product increased. Allen et al. (2018) underlined that it is apparent that nutritional beliefs are important (for example, lack of belief in nutritional benefits) to the anti-consumption decision but that there also appears to be a link between beliefs about the fat contained in dairy products, which also negatively affects dairy product consumption. Although the fat concern is evidently not the only belief about dairy product nutrition, it has received a significant focus in scientific and mainstream press for a longtime and will remain a factor in dairy anti-consumption for many years to come [49]. In addition, traditional foods, which may also include some dairy products, are a group of products that are perceived positively by consumers and, consequently, are accepted as a group of products for which health benefits can and should be enhanced [6,50,51]. Research by Clark at el. (2019) indicates that consumer preferences and a predisposition for more familiar foods, suggest that staple products would be suitable to fortify. This would act to ensure that fortified food items are consumed on a regular and sufficient basis in order to be beneficial [52]. Other research shows that, compared to the other popular snack items, encouraging the consumption of low-sugar and low-fat yogurt as a snack may provide a healthier diet [53]. Furthermore, cheese and milk are bought most frequently as part of a meal and yogurt is bought most popularly as a snack [54].

Contrastingly, in the case of opinions about the lack of food colors in the product, the problem can be relatively complex. Our research showed that the lack of food colors had rather negative associations in the consumer perception. Even if the product did not contain food colors, it may have had a negative association with the product because it was declared that products without food colors were less likely to be accepted as improving the health benefits of dairy products. Moreover, food colors may be perceived by the consumers as interfering with the naturalness of the product and, thus, it may cause some doubts among the consumers concerning the necessity of their use in food. Other studies indicate that in general consumers are more willing to accept food additives of natural origin compared with synthetic products. Moreover, it is unclear to what extent consumers are willing to sacrifice shelf life and how much they are willing to pay for additional naturalness in their food [55]. Furthermore, some consumers are afraid of popular methods applied in food processing. Research conducted by Bazhan et al. (2017) shows that consumers expressed reservations towards the consumption of low-fat milk as lacking nutritional value and the consumption of sterilized milk as containing preservatives [56]. Nevertheless, most consumers in developed countries value the naturalness of food products. They favor foods that are grown and produced by respecting nature and by using traditional methods. Furthermore, the products should be synthetic food additive-free and preservative-free. Consequently, it poses a major challenge to the food industry [55]. Another point is that, consumers may believe that adding, e.g., active ingredients demand specific technologies or even the interference with animal welfare. The impact on naturalness can be expected, since people tend to prefer natural options for ideological reasons rather than any instrumental benefit in natural products [57,58].

### 4.3. Practical Implications, Strengths and Limitations

In general, if health communications are supposed to achieve their goal and to develop more effective healthy-eating campaigns, they must challenge corporate campaigns, consider consumer understanding of food and nutrients, and address the psychology of food choice and consumption. Consumer preferences towards hedonic foods entail their attempt to find methods to include such foods in their diet. Nutrition policies should promote healthy food habits that consists in the right food choices and food substitutions, which will consequently contribute to consumer well-being [59].

The results of our research indicate that, in order to be successful on the market, entrepreneurs that are designing new food products or are introducing changes to existing products should take into account the needs of consumers with respect to increasing or decreasing the level of certain nutrients in food. It is also important to inform food business operators of the benefits perceived by consumers in relation to foods in which the levels of certain nutrients have been increased or decreased. The health benefits perceived by consumers have the potential to influence, for example, purchase and consumption intentions for selected food products, as is the case, for example, of the benefits of green pesticides which are perceived by consumers to be important in the prevention of diseases [60].

In general, the practical implications of our findings for practitioners in the dairy industry as well as for policy makers are that their efforts to influence the consumption of dairy products should include tailoring them to the specific consumers they aim to target. When designing food products, it is worth focusing on increasing the nutritional value and enhancing the health value of food that is perceived by consumers as generally possessing positive health values rather than on food that is perceived by them as possessing negative qualities. Ultimately, this may translate into an increase in consumer acceptance in relation to a specific category of food and a greater interest in health-enhancing products.

The strength of our results lies in a relatively large sample of the Polish population. Moreover, the above discussed possibilities of practical application of the findings should also be considered as the strengths of this study. The results of the research can also be used in planning and developing new products, e.g., attempts can be made to adopt the obtained research results to other industries operating in the food market. Nevertheless, the findings have some limitations. The first concerns the self-reported information obtained from the questionnaire that may be inaccurate due to the unnatural situation created by the questionnaire itself. However, the research using the real product was not logistically or economically possible in this part of study considering the size of the sample. Despite these limitations, our study provides new insights into enhancing the perceived health attributes of dairy products.

## 5. Conclusions

The results of the study can provide valuable insights for those involved not only in nutrition education but also directly for producers and processors operating on the food market. Understanding the consumers’ attention and demand for dairy products may provide benefits for manufacturers to develop new types of food and to design marketing strategies, which means developing a practical and new approach to attract consumers who want to improve their health, well-being, and quality of life.

The results obtained in the study indicated that socio-demographic characteristics of the participants, in general, were not associated with the degree of their willingness to accept the improvement of the perceived health attributes of dairy products. Overall, positive aspect perceived by consumers, connected with increasing and/or decreasing unfavorable components, plays a much greater role in their willingness to accept enhancing health values of dairy products than when compared with the influence of socio-demographic variables.

## Figures and Tables

**Table 1:** Principal component analysis (PCA) of interest in purchasing the following types of yogurt.

Item Yogurt	Factor 1 Increasing the Level of Nutrients That Positively Impact the Perception of Product’s Health Benefits (Increasing)	Factor 2 Decreasing the Levels of Nutrients That Negatively Impact the Perception of a Product’s Health Benefits (Decreasing)	Factor 3 Adding Certain Ingredients That Improve the Sensory Properties of Food Product (Adding)
with added omega-3 fatty acids	0.743		
with added coenzyme Q10	0.714		
with added zinc	0.710		
with added magnesium	0.697		
with added selenium	0.693		
with increased levels of certain vitamins and minerals to prevent deficiencies	0.678		
with added calcium	0.625		
with increased levels of substances with beneficial health effects	0.621		
with added fiber	0.608		
with information on the packaging, e.g., “High protein content”	0.581		
with information on the packaging, e.g., “Sugar-free”		0.793	
with a reduced level of fat		0.743	
with reduced levels of certain ingredients, e.g., salt or sugar, to prevent various diseases, e.g., obesity, hypertension, and diabetes		0.686	
with information on the packaging such as “Helps lower cholesterol”		0.652	
with ingredients that may cause allergies removed, e.g., milk and dairy products		0.514	
with added food colors			0.816
in which selected ingredients have been replaced with others, e.g., sugar with sweeteners			0.773
with a longer shelf life			0.731
thickened by the addition of various ingredients			0.710
percentage of explained variance (%)	42.57	11.96	5.61
Kaiser’s Measure of Sampling Adequacy (MSA): Overall MSA = 0.95			

**Table 2:** Socio-demographic characteristics of the consumers surveyed (*N* = 983, Poland) (%).

Variables	%
Gender	
Female	51.41
Male	48.59
Age	
21–27	16.30
28–34	15.99
35–44	18.18
45–54	20.06
55–64	18.81
over 64	10.66
Education	
Primary, lower secondary, vocational	47.75
Secondary	37.10
Higher	15.15
Size of the place of residence	
Village	35.86
Towns up to 20,000 residents	12.62
Towns over 20,000 residents up to 100,000 residents	19.87
Cities over 100,000 residents up to 500,000 residents	20.19
Cities over 500,000 residents	11.46

**Table 3:** Variables and their estimation properties used to build the logistic regression model.

		Unadjusted Regression Model ^1^				Adjusted Regression Model			
Variable	Level of Variable	β	e^β^	95% Wald CI	*p*-Value	β	e^β^	95% Wald CI	*p*-Value
Intercept		−4.751			<0.0001	−4.915			<0.0001
Dairy products with high health values are characterized by a low level of selected ingredients, e.g., salt, sugar, and fat	Yes	0.521	1.68	(1.09–3.32)	0.0432	0.666	1.95	(1.24–4.06)	0.0352
	No (ref.)	0	1			0	1		
Dairy products with high health benefits have high mineral and vitamin content	Yes	0.637	1.89	(1.12–3.19)	0.0169	0.627	1.87	(1.08–3.25)	0.0256
	No (ref.)	0	1			0	1		
Dairy products with high health benefits do not contain food colors	Yes	−0.341	0.71	(0.43–0.97)	0.0453	-0.275	0.76	(0.44–0.91)	0.0425
	No (ref.)	0	1			0	1		
Acceptance of nutritional improvement of dairy products	Yes	2.04	7.69	(4.48–13.21)	<0.0001	2.139	8.5	(4.75–15.18)	<0.0001
	No (ref.)	0	1			0	1		
Dairy products with high nutritional value are rich in fats	Yes	−0.86	0.42	(0.21–0.84)	0.0144	−0.974	0.38	(0.18–0.81)	0.0116
	No (ref.)	0	1			0	1		
Dairy products with high nutritional value are rich in protein	Yes	0.475	1.61	(1.05–2.73)	0.0381	0.499	1.65	(1.09–2.89)	0.0425
	No (ref.)	0	1			0	1		
Importance of increasing mineral content in dairy products		0.381	1.46	(1.25–1.71)	<0.0001	0.403	1.5	(1.26–1.78)	<0.0001
Increasing	Q2	0.529	1.70	(0.81–3.57)	0.1641	0.539	1.71	(0.78–3.75)	0.1768
	Q3	1.026	2.79	(1.34–5.83)	0.0063	1.068	2.91	(1.32–6.43)	0.0083
	Q4	1.525	4.59	(2.13–9.91)	0.0001	1.711	5.54	(2.37–12.92)	<0.0001
	Q1 (ref.)	0	1			0	1		
Decreasing	Q2	0.637	1.89	(1.09–4.00)	0.0465	0.854	2.35	(1.05–5.27)	0.0383
	Q3	0.887	2.43	(1.18–5.00)	0.0162	1.091	2.98	(1.36–6.50)	0.0061
	Q4	1.047	2.85	(1.37–5.92)	0.005	1.249	3.49	(1.58–7.72)	0.0021
	Q1 (ref.)	0	1			0	1		

^1^ The following study/article focuses on the analysis of information obtained under the unadjusted regression model; e^β^ (OR)—point estimate; β—estimate; 95% Wald CI—95% Wald confidence interval.

## Data Availability

Not applicable.

## Supplementary Materials

The following are available online at https://www.mdpi.com/article/10.3390/nu13061945/s1, Supplementary Questionnaire 1. Questionnaire used in the study (*N* = 983).
