# Supplementary material for: Directions of Changes in the Health Values of Dairy Products in the Opinion of Consumers
Source: Nutrients. 2021 Jun 5;13(6):1945. doi: 10.3390/nu13061945 (PMC8230071; doi:10.3390/nu13061945)
Supplement: Supplementary file 1 [file nutrients-13-01945-s001.zip › nutrients-1217752-supplementary.pdf]

Supplementary Materials

Directions of Changes in the Health Values of Dairy Products in the Opinion of Consumers

Marta Sajdakowska *, Jerzy Gębski and Krystyna Gutkowska

Supplementary Questionnaire 1. Questionnaire used in the study (N = 983).

1. **Do you approve of enhancing the health benefits of dairy products?**

(1) yes
(2) no

2. **Do you think it is possible to enhance the nutritional value of dairy products?**

(1) yes
(2) no

3. **Please indicate which of the given properties of dairy products you consider to be the most characteristic of dairy products with high health value. Please indicate no more than 4 answers.**

They …
(1) have a positive effect on your silhouette
(2) contain no preservatives
(3) contain the addition of live bacteria cultures
(4) have a short shelf life
(5) are little processed
(6) have low levels of selected ingredients, e.g. salt, sugar, fat
(7) have a high mineral and vitamin content
(8) are recommended by scientific institutions
(9) the packaging carries information indicating its health benefits, e.g. "Contributes to lowering cholesterol levels”
(10) contain no ingredients that may cause allergies
(11) do not contain food colors
(12) are produced with respect for the rights of farm animals

4. **Please indicate which of the given characteristics of dairy products you consider to be most characteristic of dairy products with high nutritional value. Please indicate not more than 4 answers.**

They …
(1) contain a lot of vitamins and minerals
(2) are rich in essential fatty acids
(3) contain a lot of fat
(4) contain a lot of protein
(5) contain a lot of cholesterol
(6) contain a lot of fiber
(7) contain a lot of omega-3 fatty acids
(8) contain live bacteria cultures
(9) have information on the packaging that informs about the nutritional benefits, e.g. „Rich in protein”, „With increased calcium content”, „Sugar-free”

(10) high in calories

5. Do you think that the content of the following ingredients in dairy products should be increased? Please use a scale from 1-7where 1 means “definitely should not be increased” and 7 means “definitely should be increased”

(1) minerals
(2) fiber
(3) cholesterol-lowering ingredients
(4) omega-3 fatty acid
(5) live bacteria cultures
(6) protein
(7) coenzyme Q10

6. Would you consider buying the following types of yogurt? Please use a scale from 1-7, where 1 means "definitely not" and 7 means "definitely yes"

(1) with increased levels of certain vitamins and minerals to prevent deficiencies
(2) with added food colors
(3) with added omega-3 fatty acids
(4) with information on the packaging such as "Helps lower cholesterol"
(5) with a longer shelf life
(6) with information on the package such as "Sugar-free"
(7) with ingredients that may cause allergies removed, e.g. milk and dairy products
(8) with added selenium
(9) thickened by the addition of various ingredients
(10) with reduced levels of certain ingredients, e.g. salt or sugar to prevent various diseases, e.g. obesity, hypertension, diabetes
(11) with added coenzyme Q10
(12) with added calcium
(13) with added zinc
(14) with added magnesium
(15) with increased levels of substances with beneficial health effects
(16) with a simple message on the package such as "High protein content"
(17) with a reduced level of fat
(18) in which selected ingredients have been replaced with others, e.g. sugar with sweeteners
(19) with added fiber.
